# Oxidative Stress and Mitochondrial Activation as the Main Mechanisms Underlying Graphene Toxicity against Human Cancer Cells

**DOI:** 10.1155/2016/5851035

**Published:** 2015-11-15

**Authors:** Anna Jarosz, Marta Skoda, Ilona Dudek, Dariusz Szukiewicz

**Affiliations:** Department of General & Experimental Pathology and Centre for Preclinical Research and Technology (CEPT), Medical University of Warsaw, Pawińskiego 3C, 02-106 Warsaw, Poland

## Abstract

Due to the development of nanotechnology graphene and graphene-based nanomaterials have attracted the most attention owing to their unique physical, chemical, and mechanical properties. Graphene can be applied in many fields among which biomedical applications especially diagnostics, cancer therapy, and drug delivery have been arousing a lot of interest. Therefore it is essential to understand better the graphene-cell interactions, especially toxicity and underlying mechanisms for proper use and development. This review presents the recent knowledge concerning graphene cytotoxicity and influence on different cancer cell lines.

## 1. Graphene: Properties and Applications

**Figure 1:**
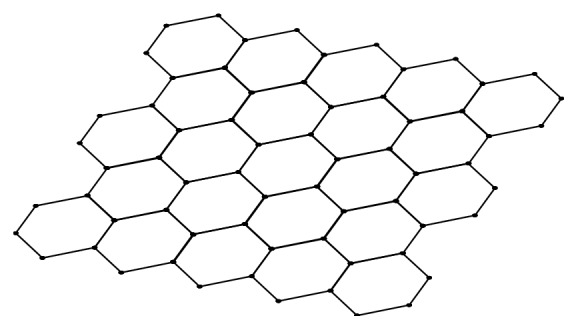
The graphene structure: single layer of sp^2^-hybridized carbon atoms arranged in 2D crystal honeycomb lattice (adapted from [9]).

Novoselov et al. first described graphene in 2004 as monocrystalline graphitic film and received Nobel Prize in 2010 for the exploration of its exceptional properties [1]. The discovery of graphene became a new driving force in the development of nanoindustry [2, 3]. Graphene is a single-atom-thick, two-dimensional sheet of sp^2^-hybridized carbon atoms arranged in a regular hexagonal pattern like in honeycomb structure (Figure 1) [4–9]. Graphene conducts heat and electricity extremely well [2] and as one of the carbon allotropes it is considered the thinnest and strongest known material [10]. The ratio of thickness of graphene sheet to the size of its surface differentiates this material from all other known nanomaterials [10]. The unique physicochemical properties of graphene are large surface area (2630 m^2^/g), extraordinary electrical (mobility of charge carriers, 200,000 cm^2^ V^−1^ s^−1^) and thermal conductivity (~5000 W/m/K), extremely high mechanical strength (Young's modulus ~1100 Gpa), and possibility of mass-production at low cost [4, 11–13]. The perfect electronic transport properties and high surface-to-volume ratios are responsible for its exceptional mechanical and rheological properties and resistance to degradation. Graphene has two active sides which are surfaces and edges that improve the attachment of biological molecules to graphene and its adhesion to the cells [11]. Graphene has higher ratio of peripheral to central carbon atoms than similar nanomaterials. Consequently atoms at the edge allow better interaction with cell membranes and interference with cell metabolism [14]. Unlike other carbon allotropes, that is, fullerenes or carbon nanotubes, graphene exhibits unique chemical and physical properties closely related to the possibility of its surface functionalization which makes it more biocompatible and less toxic [15].

Graphene and graphene-based nanomaterials are today applied in numerous fields for purposes including nanoelectronics and energy technology (supercapacitors, batteries, composite materials, transistors, solar cells, fuel cells, matrix for mass spectra, and hydrogen storage), energy storage, sensors, catalysis, and biomedicine [2, 4, 11, 12]. Due to their unique mechanical properties, such as high elasticity, flexibility, and adaptability for tissue engineering graphene family nanomaterials (GFNs) have been investigated in several biomedical applications especially cancer therapy, drug delivery, and diagnosis [5, 16, 17]. Other biomedical applications comprise gene delivery, antibacterial and antiviral materials, tissue engineering, and biocompatible scaffolds for cell cultures. Graphene-based materials are promising in the field of biosensing and bioimaging (optical sensing, fluorescence imaging probes, and electrochemical sensing) [4, 5, 12, 18]. Furthermore, graphene nanomaterials have been used in advanced therapeutic techniques such as photothermal and photodynamic therapies [3, 16].

Graphene and its derivatives, referred to as graphene family nanomaterials (GFNs), include graphene oxide (GO), its reduced form (rGO) and single- or few-layer graphene, graphene nanosheets (GNS), and graphene nanoribbons [4, 11, 19]. Graphene nanoparticles, depending on the method of synthesis, can show different morphologies and chemical or physical properties [20]. So far various approaches have been developed to synthesize graphene and its derivatives such as mechanical exfoliation, epitaxial growth, or unzipping carbon nanotubes. The mechanical exfoliation, firstly used by Novoselov in 2004, resulted in few-layer graphene from highly oriented pyrolytic graphite. Graphene samples with the lateral size up to millimeter-range were obtained after many method modifications but still are too large and cannot be produced on a large scale, hence the inability to be used in most practical applications. Chemical vapor deposition (CVD) based on dissolving carbon atoms into a metal substrate allows producing large scale graphene films. Graphene nanoribbons (GNRs) of precise dimensions and 100% yield can be obtained by the novel strategy based on longitudinal unzipping carbon nanotubes. However, the most developed method for the mass-production of graphene is the exfoliation of graphene oxide (GO). Oxygen functional groups on the graphene surface make GO and rGO sheets strongly hydrophobic although the electrical conductivity is lower than that of pristine graphene. Poor conductivity can be bypassed in the process of liquid phase exfoliation of graphite where high-quality monolayer graphene at significant yield can be produced [15]. In our previous article we have described numerous methods of graphene synthesis related with the development of various forms of graphene which differ in the quality, number of layers, and the amount of the structure defects [21]. Lots of the possible applications of graphene derivatives obtained in different conditions make it problematic to use graphene safely in biomedicine or tissue engineering. In this paper we have focused on the impact of graphene family nanomaterials (GFNs) on the different cancer cells, the possible mechanisms of graphene toxicity, and available applications of graphene in cancer therapy or drug delivery.

## 2. Graphene Family Nanomaterials (GFNs)

Among other members of graphene family nanomaterials (GFNs) graphene oxide (GO) is one of the most important chemical graphene derivatives. GO is a highly oxidized form of graphene [4, 22, 23] produced mainly by chemical methods through energetic oxidation of graphite using different oxidant agents or known procedures as in Hummers method [10, 12]. GO nanosheets present hydroxyl and epoxide functional groups on their basal surface and carboxyl functional groups on their plane edges [3]. GO has usually 1–3 layers (1-2 nm thick), with size ranging from a few to several hundred nanometers [12]. GO is hydrophilic and forms stable suspensions in pure water but in salt and other biological solutions it creates aggregates [24, 25]. Reactive COOH and OH groups in GO facilitate connection with various materials, such as polymers, biomolecules, DNA, protein, quantum dots, or Fe_3_O_4_ nanoparticles which improve the solubility and prevent aggregation in salt-containing physiological buffers [3, 12].

Improved properties of graphene oxide make it useful in biological and medical applications, as a surface coating material for implants and also as a stimulator of growth and differentiation of the cells [12, 17, 18]. The large aromatic surface of graphene oxide with lots of functional groups allows adsorbing molecules with high affinity and creating stable complexes which make GO an ideal nanocarrier for effective drug and gene delivery [23, 26]. Different targeting molecules such as folic acid or antibodies can be conveniently immobilized on GO which allows precise and efficient delivery of GO into targeted cells. Solid tumor cells are more acidic (pH ~ 6.8) than normal cells (pH 7.4) and are ideal candidates for controlled release of anticancer drugs [27]. Lowered pH in some drug molecules additionally increases their solubility and decreases their tendency to stay adsorbed which eventually leads to the controlled endocytosis and the release in lysosomes [15]. pH-responsive and integrin *α*_v_ *β*_3_ monoclonal antibody functionalized graphene oxide is an example of the nanocarrier for targeted delivery and controlled release of doxorubicin (DOX) into cancer cells [27].

Reduced graphene oxide (rGO) is the product of thermal or chemical modification of graphene oxide (GO) with reducing agents (e.g., hydrazine) [3, 4]. rGO possess lower number of oxygen containing functional groups than GO [28]. The reducing conditions greatly influence the properties of GO such as electrical conductivity, surface charge, or water dispersibility (increase hydrophobicity) [4]. rGO possesses high capacity for hydrophobic interactions among various functional molecules but it leads to creation of aggregates with weak stability under physiological conditions. Surface modification of rGO with polymers or biopolymers has been used to stabilize and improve the properties of rGO and use it as a nanocarrier [29].

Graphene platelets (GPs) are produced by physical methods directly by exfoliation of graphite without the initial stage of oxidation. GPs are hydrophobic and form stable hydrocolloids [10]. Zero-dimensional, single-atom layer graphene quantum dots (GQDs) have lateral dimensions below 100 nm and size of 10 nm or less [16]. GQDs are biocompatible due to their small size and high oxygen content which improves solubility and stability in water or serum [12, 16]. Graphene quantum dots due to their excellent photoluminescent properties are promising agents for optical probes in bioimaging [6]. Graphene nanoparticles, referred to as graphene nanoribbons, are formed by the longitudinal unzipping of multiwalled carbon nanotubes [30].

## 3. Graphene and Cells

**Figure 2:**
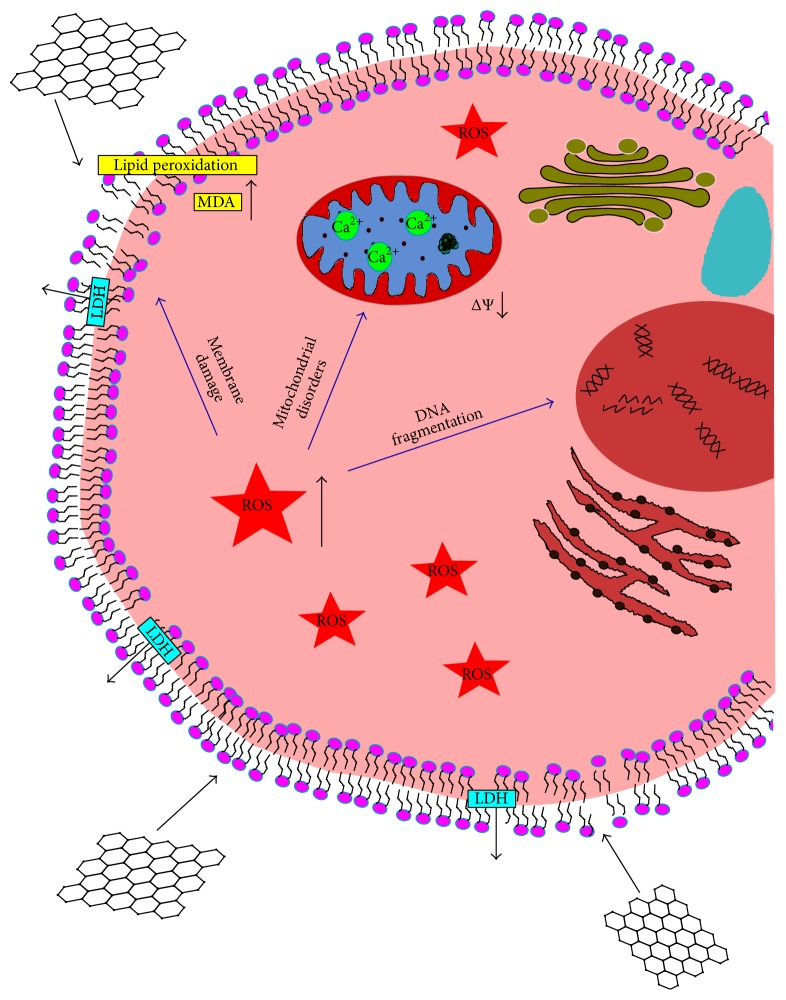
Schematic toxicity mechanisms of graphene on human cancer cells. Graphene provides the formation of reactive oxygen species (ROS) which are the cause of DNA (fragmentation and condensation) and cell membrane damage (release of LDH, lipid peroxidation, and increase in MDA-malondialdehyde), mitochondrial disorders (reduction of mitochondrial membrane potential ΔΨ, increase in Ca^2+^), and cell death.

The potential toxic effects of graphene materials on the environment and on the human health have recently attracted considerable attention among researchers. Understanding of the interactions of GFNs with living systems and their adverse effects in vitro and in vivo is essential for further development and safe use of graphene-based nanomaterials [11]. Cytotoxicity studies of graphene include the influence on the cell viability and morphology, membrane integrity, ROS generation, DNA damage, gene expression, DNA damage, and mechanism of uptake (Figure 2) [4, 10, 13]. The interactions of graphene nanoparticles with the cells depend on the physicochemical and electrical properties [5, 12, 40–43]. The reports indicate that morphology (size, shape, and sharp edges), surface charge, surface functionalization, dispersibility, state of aggregation, number of layers, purity, and method of synthesis (e.g., CVD [21], arc-discharge [30], and biological methods [31]) are the key factors that influence the mechanism of uptake (passive diffusion and endosomal uptake) and tissue response to graphene-based nanomaterials [2, 4, 5, 20, 30]. Moreover, the toxic effect of graphene highly depends on the conditions of the experiment, which include the time of exposure, dose, type of the cells, and the method used to establish the cell viability [19, 24, 30, 31, 33].

The chemical methods used in the production of graphene nanomaterials including oxidation or reduction of graphene oxide bring harsh conditions and toxic agents, such as hydrazine or its derivatives, which influence the structure of graphene and its safety. One of the approaches used to decrease the toxicity of graphene involves aqueous and environmentally friendly reduction strategy based on bacterial and yeast respiration [31]. Recently used microbial biomass for the reduction of GO including* Escherichia coli* [44],* Bacillus marisflavi* [31], and* Ganoderma* extract [5] has significantly increased biocompatibility of graphene.

The majority of GFNs have poor solubility and create aggregates in salt-containing physiological buffers due to electrostatic charge and nonspecific binding to proteins [11]. Functionalization of pristine graphene via covalent or noncovalent coatings by various materials such as polymers, DNA, proteins, and nanoparticles greatly improves the biocompatibility [3]. Surface modifications of graphene nanomaterials also improve their solubility and significantly reduce toxic interactions with living systems. Significant changes in biocompatibility have been achieved by producing graphene reinforced composite materials with polyethylene glycol (PEG) or other biopolymers such as chitosan, hyaluronan (HA), or dextran [3, 11, 13, 17, 19].

Most of the members of graphene family nanomaterials easily enter the living cells because of the small size, sharp edges and rough surface [11]. Additionally, negatively charged (−30.89 eV) GO can easily accumulate inside the cell [40]. The uptake can be also affected by the shape and the aggregation state of GO sheets [35]. The presence of carboxyl, epoxy, and hydroxyl groups in GO reduces its cytotoxicity [40] and the small size (smaller than 5 nm) and the high content of oxygen improve the solubility and increase biocompatibility [16]. However, the mechanism of cellular uptake and the fate of graphene inside the living cells are still not fully understood. This process may depend on the cell type, on the properties of graphene, or on both of these factors. Some researchers suggest endocytosis as a basic mechanism of cellular uptake for PEG-GO while others combine endocytosis and macropinocytosis depending on the formation of smaller or larger aggregates of PEG-graphene nanoribbons [3].

The physical interactions of graphene with the cell membranes are one of the major causes of GFNs cytotoxicity [5, 19, 45]. Hydrophobic forms of graphene interact with the cell membrane lipids [19] while the other forms may bond to the cell receptors and interfere with the cell metabolism, inhibit nutrient supply, and induce stress or cell death [10]. Moreover, graphene itself can bind the micronutrients and amino acids from the cell culture medium which limits their availability and inhibits cellular growth and viability [24]. GO is smaller and less toxic than rGO because of the high oxygen content, smoother edges, and hydrophilic properties. Reduced graphene oxide has high affinity to the cell membranes and the irregular and sharp edges affect their integrity, stimulate receptors, and activate mitochondrial pathways which may cause apoptosis [46].

Oxidative stress and generation of reactive oxygen species (ROS) can be involved in the toxic effects of graphene-based nanomaterials [16, 19, 45]. When the cell homeostasis is disrupted and the enzymes responsible for reducing ROS (superoxide dismutase and glutathione peroxidase) fail, the macromolecules, such as proteins, DNA, and lipids, can be damaged, which greatly influence the cell metabolism and signaling [19, 42]. The interactions of the GO with the cells can lead to excessive ROS generation, which is the first step in the mechanisms of carcinogenesis, ageing, and mutagenesis [22].

Except for the plasma membrane damage and oxidative stress induction graphene can cause apoptosis and/or cell necrosis through the direct influence on the cell DNA or mitochondrial activity [18]. Graphene nanoparticles can induce dissipation of the mitochondrial membrane potential which subsequently increases the generation of intracellular ROS and eventually triggers apoptosis by activating the mitochondrial pathway [31]. The interactions of graphene with cell genetic material are based on DNA-intercalation and cleavage mechanisms [13]. Difference in the structure of rGO and GO makes rGO more potent to penetrate cell compartments and directly interact with the nuclear DNA resulting in genotoxic effects [46].

Additionally, graphene can directly interact with different genes encoding important proteins and enzymes [4, 13]. Other indirect mechanisms of GO cytotoxicity involve DNA damage caused by ROS [13], inhibition or activation of specific enzymes [22], or reaction with other cell components such as proteins and polysaccharides [13]. For better understanding of the mechanisms of graphene action inside the cell further studies are required, particularly to explain the cellular interactions of graphene materials with proteins and cell membrane lipids on a molecular level [19].

### 3.1. Breast Cancer Cell

Many of the currently available methods for producing graphene are not environmentally friendly and rGO obtained by these methods is not safe enough to use in biological and medical applications. Therefore researchers developed a novel and simple approach for rGO synthesis using microorganisms which is cost-effective and safe for the environment. Gurunathan et al. compared the cytotoxicity of GO obtained from graphite powder using a modified version of Hummers and Offeman's method with rGO synthesized by* Bacillus marisflavi* biomass on human breast adenocarcinoma cells (MCF-7) using WST-8 assay. Incubation of MCF-7 cells with both B-rGO (biogenic rGO) and GO at concentrations ranging from 0 to 100 *μ*g/mL showed dose-dependent graphene cytotoxicity. In concentrations higher than 60 *μ*g/mL graphene markedly decreased the cell viability and increased ROS generation and release of LDH. Surprisingly, bacterial rGO had stronger cytotoxic effect on MCG-7 cells compared to GO [31]. In another experiment Gurunathan and colleagues used mushroom extracts (*Ganoderma*) to reduce graphene oxide. They examined the influence of GO and GE-rGO on MDA-MB-231 human breast cancer cells using WST-8 viability assay, membrane integrity test (LDH assay), and DCFH-DA assay as a quantitative method for oxidative stress assessment. The cytotoxicity of graphene was dose-dependent (0–150 *μ*g/mL) especially at the higher concentrations where elevated levels of ROS induced membrane damage and LDH leakage in the presence of GE-rGO [5]. These studies indicate that rGO synthesis with the use of bacteria and fungi is easier, less expensive and works better for the development of a potential therapeutic agent that targets breast cancer cells.

In vitro anticancer activity of GO was examined in various concentrations (10, 20, 40, and 80 *μ*g/mL) on human breast cancer cells MCF-7 using MTT viability assay. GO showed approximately 13% inhibition of cell viability of MCF-7 cells and the cytotoxicity at dose-dependent manner [32]. Other tests concerning cytotoxicity of GO were carried on human adenocarcinoma breast cancer cells (MDA-MB-231) using Cell Counting Kit-8 (CCK-8) assay. 48 h incubation with GO in concentrations ranging from 100 *μ*g/mL to 500 *μ*g/mL showed increasing cytotoxicity against MDA-MB-231 cells together with the increasing amount of graphene in the medium. Further studies showed that GO reacts directly with genomic DNA and inhibits cell replication with complete blockage of human glyceraldehyde-3-phosphate dehydrogenase (hGAPDH) gene at the concentration of 1 *μ*g/mL. MDA-MB-231 cells treated with GO even at low concentration (10 *μ*g/mL) after 24 h incubation showed signs of apoptosis. Hence, scientists tested 30,000 genes to examine the impact of GO on the gene expression at the cellular level. The results revealed 101 genes (mainly responsible for DNA-damage control, cell apoptosis, cell cycle, and metabolism) that showed 2-fold or even greater expression changes after GO treatment at the concentrations of 10 *μ*g/mL and 100 *μ*g/mL. Additionally, GO increased expression of ATM and Rad51 genes (DNA repair proteins) which can explain the influence of graphene on the cell DNA [13].

Zhou and coworkers evaluated the cytotoxicity of GO modified with polyethylene glycol (PEG) using three cell lines derived from human breast cancers: MDA-MB-231, MDA-MB-436, and SK-BR-3. PEG-GO had no apparent influence on the cell viability but inhibited cancer cell migration and invasion. PEG-GO disrupted F-actin filaments responsible for cell migration by depleting ATP levels through downregulation of mitochondrial energy metabolism [47]. Another research on the human breast cancer cells MDA-MB-231 with pristine graphene and graphene oxide also showed no apparent influence on the cell viability at low concentrations but prominent inhibition of migration and invasion [48].

Recent findings have proved that the functionalization of the graphene surface makes it less toxic. Mullick Chowdhury et al. investigated the cytotoxicity of oxidized-graphene nanoribbons coated with the amphiphilic polymer PEG-DSPE (O-GNR-PEG-DSPE) at various concentrations (0–400 *μ*g/mL) on Sloan Kettering breast cancer (SKBR3) cells and Michigan Cancer Foundation-7 (MCF-7) breast cancer cells using Alamar blue assay. Both cell lines showed the reduction in viability by about 10%–15% at the highest concentrations after 24 h incubation with the copolymer. SKBR3 cells incubated with O-GNR-PEG-DSPE demonstrated slight increase in the LDH release while MCF-7 cells did not show any statistically significant LDH leakage. Additionally, SKBR3 and MCF-7 cells showed small or no uptake of O-GNR-PEG-DSPE. The results indicate that graphene copolymer has no toxic effect on the tested cells up to 10 *μ*g/mL and exhibits low cytotoxicity even at the highest concentrations (400 *μ*g/mL) [30].

The toxicity of covalently pegylated nano-GO with unmodified rGO was compared using MTS assay and MCF-7 human epithelial breast cancer cells. The half maximal inhibitory concentration (IC50) of nano-rGO was established at the concentration of approximately 80 mg/L, while for pegylated nano-GO it was at about 99 mg/L [32]. According to MTT assay fluorinated form of graphene oxide (FGO) even at the concentration of 576 *μ*g/mL showed no toxicity to human breast cancer cells (MCF-7) [49]. Waiwijit and coworkers investigated the toxicity of graphene-carbon paste (GCP) in four different concentrations (1, 2.5, 5, and 10 wt%) on MDA-MB-231 breast cancer cells also using MTT assay. The cell viability decreased after longer incubation periods (48 and 72 h) and at the presence of the highest concentration of GCP in comparison to the cultures with CP alone. Moreover, MDA-MB-231 cancer cells exhibited increased ROS generation with the increasing time of incubation and the amount of GCP in the culture medium [42]. Together, these studies demonstrate the different impact of graphene nanomaterials on breast cancer cells including the cell viability and cytotoxicity connected with the generation of ROS, loss of the membrane integrity, and DNA damage which may have potential clinical advantage pertaining to increased therapeutic efficacy and decreased local toxicity of the used nanomaterial.

### 3.2. Cervical Cancer Cell

Remarkably durable and prolific HeLa cells derived from cervical cancer are more sensitive to the graphene than other cell lines. According to Zhang et al. GO showed high cytotoxicity to HeLa cells even at low concentrations. The biological responses induced by GO were evaluated by series of assays, including MTT, malondialdehyde (MDA), superoxide dismutase (SOD), lactate dehydrogenase (LDH), and reactive oxygen species (ROS). HeLa cells were treated with different concentrations of GO ranging from 0 to 80 *μ*g/mL and cultured for 3 h and 24 h. MTT test results showed dose-dependent GO cytotoxicity with the cell viability at about 50% at the concentration of 80 *μ*g/mL. To evaluate the lipid peroxidation and oxidant stress the levels of MDA and SOD enzyme activity were measured in the cell lysates. The results showed an obvious increase in MDA production after exposure to 80 *μ*g/mL of GO and decreased SOD activity. Moreover, the incubation of HeLa cells with 80 *μ*g/mL of GO for 24 h increased the levels of ROS 17 times. The researchers suggested that the cytotoxicity of GO is not associated with the cell uptake [33].

Instead of the biological assays measuring cell activity and viability there are available more selective, more sensitive, and faster electrochemical approaches to evaluate the toxicity of graphene. Yoon et al. used cell-based electrochemical impedance biosensing with interdigitated indium tin oxide (ITO) electrodes to analyze toxicity of graphene nanoflakes in HeLa cells. Researchers used two different sizes of graphene flakes (80 nm and 30 nm) in the concentration of 400 *μ*g/mL and monitored the cytotoxicity for 1 day. The studies showed greater cytotoxic effect of the smaller 30 nm graphene nanoflakes due to their higher uptake, while 80 nm graphene nanoflakes agglomerated on cell membranes causing less harm to the cells [34].

Liu's group conjugated graphene oxide with dextran, a widely used surface coating biopolymer. They cultured HeLa cells with different concentrations (10, 50, and 200 mg/L) of GO and GO-DEX and studied in vitro toxicity for 24 h, 48 h, and 72 h. The cell counting data showed dose-dependent decrease in the cell proliferation after incubation with GO and notably smaller influence on the cell count after GO-DEX treatment. The calcein AM/propidium iodide (PI) staining was carried out to further determine graphene toxicity. The results revealed that GO did not induce significant cell death even at high concentrations up to 200 mg/L, while GO-DEX showed no influence on the cell growth and viability. All the evidence demonstrates that dextran coating may improve the biocompatibility of GO [50].

In other studies the cytotoxicity of graphene polymer (GQD-PEG) was evaluated on HeLa cells using WST-1 assay. GQD-PEG did not induce apoptosis or necrosis even at the concentration of 160 *μ*g/mL. LDH release and ROS level measurements showed no impact of GQD-PEG on the cell membrane integrity and oxidative stress generation probably because of the small size of the particles (smaller than 5 nm) and the presence of PEG polymer [16]. However, HeLa cells showed a reduction of the cell viability by 60% after 24 h incubation with 400 *μ*g/mL O-GNR-PEG-DSPE (oxidized-graphene nanoribbons (O-GNRs) with the amphiphilic polymer). As the dose increased, the survival rate of the cells decreased together with the release of LDH. On the images of the cells lots of swollen intracellular vesicles were observed together with disrupted plasma membranes which are a characteristic feature in necrotic cells [30].

### 3.3. Lung Cancer Cell

The biological effect of GFNs on lung cancer cells depends mainly on the size and concentration of graphene [22, 35]. Hu et al. investigated the cellular effect of different concentrations (0 to 100 *μ*g/mL) of GO nanosheets on human alveolar adenocarcinoma cell line (A549). MTT assay showed concentration-dependent cytotoxicity and about 50% decrease in cell viability after incubation with GO at the concentration of 100 *μ*g/mL. Interestingly, the cell viability was greatly mitigated after addition of 10% FBS (fetal bovine serum) into the culture medium. TEM imaging demonstrates that precoating of GO with FBS prevents cell membranes from the damage, the outflow of cytoplasm, and eventually cell death. GO nanosheets possess high adsorption capability for proteins in the medium and therefore cytotoxic effect of GO precoated with 10% FBS was largely reduced [36].

In other experiments scientists compared the cytotoxicity of GO nanosheets and reduced with hydrazine rGO nanosheets characterized by lower thickness and less surface defects. The metabolic activity assays based on succinate dehydrogenase activity in the mitochondria showed that GO in the concentration of 20 *μ*g/mL slightly influenced the viability of A549 cells (20%) but in higher concentration (85 *μ*g/mL) reduced the cell viability to 50% within 24 h. rGO nanosheets reduced the A549 cell viability to 47% and 15% with 20 and 85 *μ*g/mL, respectively. Therefore, rGO nanosheets are significantly more cytotoxic than GO's which is because of different surface charge and functional groups on the nanosheet surfaces. Transmission electron microscopy (TEM) showed that graphene nanosheets could be internalized within A549 cells via endocytosis. However, flow cytometric analysis demonstrated no apoptosis in A549 cells treated with GO nanosheets (20 and 85 *μ*g/mL for 24 h) but cell cycle arrest in the G2 phase (mitosis metaphase). These data suggest that the observed small decrease in the cell viability is not because of the cell death but rather might arise from GO-retarded cell cycle which restrains the proliferation rate [37].

The group of scientists investigated also the cytotoxicity of graphene oxide (GO) and highly hydrogenated graphene (HHG) in concentrations that ranged from 3.125 *μ*g/mL to 400 *μ*g/mL. The results from MTT and WST-8 assays indicated that HHG was more toxic to A549 cells than GO and that the toxicity was dose-dependent. The percentage of viable cells after 24 h treatment with GO and HHG in the concentration of 400 *μ*g/mL was 43% and 26%, respectively [24].

In contrast, Chang et al. reported that graphene oxide (GO) is a reasonably safe material at the cellular level. Researchers examined the toxicity of GO at the concentration range from 0 to 200 *μ*g/mL on human lung carcinoma epithelial cell line A549. In this comprehensive study the morphology, viability, apoptosis, ROS production, and membrane integrity were examined. The CCK-8 assay used to estimate the GO toxicity showed dose- and size-dependent loss of the viability with little influence of the culture period. However, the level of apoptosis was not relevant to the dose or the size of the GO samples and exposure to GO did not induce LDH leakage. The LDH levels of GO-treated cells (for 200 *μ*g/mL was 6%) were even slightly lower than those of the control cells (7.5%). GO induced oxidative stress in A549 cells even at low concentrations, but with no obvious toxicity. The results showed that the cells grow on the GO films very well and there is no considerable difference in the morphology and density of the GO-treated and control cells. There is no impact on the ultrastructure of A549 cells and no signs of GO sheets inside the cells. These results indicate that GO is biocompatible and has a great potential for being the substrate for the cell growth [35].

de Marzi et al. with the same cell line investigated the impact of graphene oxide on the viability using MTT assay. Graphene oxide was used at various concentrations (10, 50, and 100 *μ*g/mL) and in two different flake sizes (1.32 *μ*m and 130 nm). The results showed slight loss in the viability of the A549 cells after 24 h incubation with both types of GO. The comet assay showed size-dependent genotoxic effect on the cells with high degree of toxicity even at the low concentrations with 130 nm GO flakes [22]. Cytotoxicity and distribution of GO inside the A549 cells were evaluated by Jin and coworkers using CCK-8 assay and transmission electron microscopy (TEM), respectively. After 4 h incubation with GO in concentrations of 100 and 300 *μ*g/mL there was no significant decrease in the cell viability. GO was present inside the cells in the cytoplasm and nucleus but cellular organelles were not affected [40].

Yuan et al. examined the cytotoxicity of graphene quantum dots (GQDs) with various surface modifications (NH_2_, COOH, and CO-N (CH_3_)_2_) in human lung carcinoma cells (A549 cells) using MTT assay. GQDs with different functional groups had low cytotoxicity even when the concentration reached 200 *μ*g/mL. Moreover, the three kinds of GQDs did not induce cell apoptosis and/or necrosis. GQDs (50 *μ*g/mL) were localized in the cytoplasm and did not enter into the cell nucleus. GQDs are smaller and provide less damage to cell membranes than GO and therefore are more biocompatible and less cytotoxic to cells even when modified with different chemical groups [6]. Other studies showed that pegylated graphene quantum dots (GQDs-PEG) are practically not toxic to A549 cells at all [16]. The inconsistency of these results might come from the different methods of preparation or synthesis of GO and distinct testing models.

### 3.4. Liver Cancer Cell

The increasing number of possible applications of graphene nanomaterials triggers considerable concerns about the impact on health and environment though further more thorough investigations are vital. Chatterjee et al. investigated toxicity of various concentrations of graphene oxide (GO) and reduced graphene oxide (rGO) on HepG2 cells for 24 h. According to EZ-Cytox assay the cells viability was clearly dose- and time-dependent for both nanomaterials but rGO indicated higher cytotoxicity with unclear converse change after 16 h of exposure. EC20 and EC50 for rGO were 8 mg/L and 46 mg/L, respectively, whereas they were 10 mg/L and 81 mg/L for GO. The microscopic images showed increased internal granularity of the GO-treated cells which indicates that GO was internalized by HepG2 cells through endocytosis. The rGO treated cells showed outsized aggregation and accumulation of rGO on the cell membrane due to its hydrophobic nature. Difference in the uptake efficiency explains various modes of cytotoxicity [4].

One of the principal mechanisms underlying nanomaterial toxicity involves oxidative stress. In the experiments both GO and rGO induced release of reactive oxygen species (ROS) in HepG2 at dose-dependent manner. However, rGO mediated ROS production was the result of physical interaction while oxidative stress induced by GO involved NADPH oxidase and significant increase in the antioxidative enzyme genes (SOD1, SOD2, CAT, GSTA1, and GSTA4) expression. The toxicity of graphene can also be caused by direct interaction with the cell DNA. GO and rGO induced both single and double stranded DNA damage. rGO did not significantly influence the DNA repair gene expression and DNA damage resulted from physical interactions rather than biological one. Moreover, GO and rGO both caused increase in the apoptosis rate of HepG2 cells. However, apoptosis induced by GO was dose- and time-dependent and involved alterations in expression of the key apoptotic genes whereas rGO elicited apoptosis only at lower dose and early time of exposure. The cytotoxicity of rGO is probably caused by the strong hydrophobic interactions with the cell membranes and eventual destruction by extremely sharp edges and highly depends on their uptake by HepG2 cells [4].

The objective of the study of Lammel and his coworkers was to evaluate the cytotoxicity and underlying mechanism of two different graphene derivatives: graphene oxide (GO) and carboxyl graphene (CXYG) towards human hepatoma cell line. It was observed that cells exposed to GO and CXYG in concentrations of 16 *μ*g/mL for 24 h were completely covered with the nanomaterial and further increase in the concentration caused unspecific cell damage due to mechanical stress. TEM and scanning electron micrographs demonstrated that both GO and CXYG were able to penetrate the plasma membrane and cumulate in the intracellular vesicles resulting in altered cell morphology and an augmented number of apoptotic cells. Exposure of HepG2 to GO (1–16 *μ*g/mL) and CXYG (2–32 *μ*g/mL) for 72 h caused dose-dependent increase in the fluorescence intensity indicating an elevated metabolic activity of the cells which suggests plasma membrane damage. Loss of the membrane integrity was associated with a strong physical interaction of GO with the phospholipid bilayer and increased metabolism was probably associated with energy-dependent process involved in plasma membrane repair. Elevated fluorescence intensity at the high exposure concentrations can be also explained by oxidative stress increase. However, the underlying ROS-generating mechanisms were distinct after GO and CXYG treatment. Exposure to GO and GXVG indicates mitochondrial membrane depolarization and/or a decrease in the amount of mitochondria which leads to increased intracellular ROS. The authors concluded that plasma membrane damage and oxidative stress are the key factors in graphene-induced cytotoxicity of HepG2 cells [18].

Yuan et al. applied the iTRAQ-coupled 2D LC-MS/MS approach to analyze the protein profile change of HepG2 cells treated with graphene oxide. They observed only a moderate variation of protein levels within the cells [45, 51]. Moreover, MTT assay resulted in 17% loss of the cell viability in the cells treated with GO [45].

### 3.5. Nerve Cell Cancer

Graphene toxicity and biocompatibility were further established by Jaworski et al. who examined the influence of graphene platelets (GPs) on two different human glioma cell lines (U87 and U118) with high degree of malignancy. The GP-treated cells were more oval and denser and in both cases graphene platelets created agglomerates close to the cell bodies but did not enter the cells. GPs caused cell membrane disruption higher in U87 than in U118 cells. Exposure to graphene at the concentration of 100 *μ*g/mL for 24 h resulted in 54% and 58% decrease in the cell viability in U87 and U118 cells, respectively. The degree of apoptosis was higher in both glioma cell lines (68% in U87 and 99% in U118) together with necrosis present only in U87 (24%). The results indicate that the high concentration and the direct physical contact with the cells are the main cause of graphene toxicity. Difference in the activity of genes involved in a cell cycle regulation of the U87 and U118 cells is responsible for the susceptibility to programmed cell death indicating the potential applicability of GP in anticancer therapy [10]. Similar results of nano-rGO were obtained in U87MG glioblastoma cell line using MTS assay where half maximal inhibitory concentration (IC50) reached 85 mg/L [52]. Jaworski et al. using the same glioma cells (U87 and U118) as previously mentioned investigated cyto- and genotoxicity of GO and rGO platelets. In vitro analysis showed that both GO and rGO enter glioma cells and reduce the cell viability and the proliferation with increasing doses. However, the lower cell vitality and the higher degree of apoptosis were observed after rGO treatment which indicates that GO is less toxic to glioma cells than rGO [14]. The scope of another experimental in vitro study on glioblastoma cancer cells U87 was to determine the cell viability and DNA fragmentation after exposure to different carbon allotropes. All studied nanoparticles did not alter the cell morphology; however pristine graphene (GN) and reduced graphene oxide (rGO) led to a significant decrease in the cell viability. The comet assay results demonstrated that DNA damage was caused by GN, rGO, graphite, and ultradispersed detonation diamond (UDD) and only GO had no genotoxic effect on U87 cells. These findings indicate the potential use of GO as a drug nanocarrier and GN, rGO, graphite, and UDD in the direct elimination of glioblastoma multiforme cells because of their higher toxicity [46].

Moore and coworkers investigated the impact of nanographene (nGr) in U-138 glioblastoma cells. Cytotoxicity was measured in vitro using PrestoBlue cell viability assay after 24 h incubation. The results showed significant increase in the number of dead cells and the decrease in cell density after graphene treatment in the concentrations higher than 50 *μ*g/mL [17].

Yuan et al. examined the cytotoxicity of graphene quantum dots (GQDs) with different surface modifications (NH_2_, COOH, and CO-N (CH_3_)_2_) in human neural glioma cells (C6) using MTT assay. Conversely, data analysis showed low cytotoxicity and good biocompatibility for all tested graphene nanomaterials even at the very high concentrations (200 *μ*g/mL) [6]. Carboxylated graphene oxide (GO-COOH) and chlorotoxin-conjugated graphene oxide (CTX-GO) both had negligible toxic effects on C6 cells (80% of viability at concentrations of 3.0 *μ*g/mL, 7.5 *μ*g/mL, and 15.0 *μ*g/mL) [23]. Coating graphene with the multifunctional PLA-PEG (poly(lactide) and poly(ethylene glycol)) reduced the toxicity of uncoated graphene and did not show signs of dose-dependent toxicity up to 250 *μ*g/mL [17].

Interesting results were obtained by Oh et al. Scientists used MTT assay to examine the viability of SH-SY5Y cell line grown on partially functionalized graphene sheets with oxygen or fluorine. SH-SY5Y cells cultured on the oxygenated graphene sheets showed approximately 138% viability but only 50% viability on the fluorinated graphene compared to pristine graphene samples. The increase in cell proliferation can be explained by adhesion of the hydrophilic oxygenated graphene sheets to the cell surface [8].

### 3.6. Other Cancer Cells

Except described cancer cell lines where cytotoxic effect was predominant, some reports show only slight decrease in the cell viability with improved influence of graphene on the cell proliferation and survival [22, 25, 53]. The cytotoxicity of graphene depends on various possible mechanisms including interactions with the cells or culture medium. de Marzi et al. using graphene oxide at growing concentrations (10, 50, and 100 *μ*g/mL) and in two different flake sizes (1320 nm and 130 nm) investigated the cytotoxic effect on CaCo2 human colorectal adenocarcinoma cell line. Both micro- and nano-GO exhibited high biocompatibility and increased CaCo2 cell proliferation slightly decreasing with higher concentrations of nano-GO. The 24 h comet assay showed that micro-GO flakes genotoxicity rose together with the used concentration, while nano-GO had no significant genotoxic effect on treated cells [22].

Beyond exerting little cytotoxic effects on the cells, Ruiz et al. observed morphological changes, cell enlargement, and better attachment to GO-coated slides of HT-29 mammalian colorectal adenocarcinoma cells (control glass slides and glass slides coated with 10 *μ*g of GO). The results indicated promotion of mammalian cell proliferation, spreading, and growth after graphene oxide exposure [53].

Wu et al. evaluated the cytotoxicity of graphene oxide (GO) on human multiple myeloma cells (RPMI-8226). Increasing GO concentration from 10 to 100 mg/L after 24 h treatment reduced the cell viability from 95.6% to 79.6%, respectively. Cells treated with GO were round with little cell shrinkage but with no typical apoptotic features. Annexin V-FITC/PI staining by flow cytometry showed no significant differences in the cell apoptotic rate between the untreated and GO-treated cells suggesting only slight cytotoxicity of GO [38].

Sun and his group examined toxicity of single-layer pegylated graphene oxide sheets (NGO-PEG) soluble in buffers and serum. Incubation of Raji cells (Burkitt's lymphoma B lymphocytes) in various concentrations of NGO-PEG for 72 h showed no obvious toxicity except a slight delay of the cell growth at the highest concentration (150 mg/L) [25].

Human prostate cancer cells (PC3) were incubated in the presence of different concentrations (0–180 *μ*g/*μ*L) of chemically reduced graphene oxide (CRGO) and chitosan magnetic graphene nanoparticles (CMG) for 72 hours. The cytotoxicity was evaluated using the WST-1 assay and the results revealed dose-dependent increase in graphene oxide cytotoxicity while CMG nanoparticles did not show any toxicity at all the tested concentrations. Chitosan-coated graphene oxide is soluble in both organic and acidic aqueous solutions and less toxic than nonfunctionalized GO and hence has higher therapeutic efficacy [39]. New insights into specific cancer treatment were presented in the research on metastasis of prostate cancer cells PC3. With the low influence on the cell viability pristine graphene and GO effectively inhibited migration and invasion of these cancer cells with no apparent effect on the induction of apoptosis [48].

Conventional therapeutic approaches to eradicate all cancer cells fail because of the presence of tumor-initiating cells that are resistant to drugs, chemotherapy, and radiation. Cancer stem cells (CSCs) constitute a minority of the overall cancer cell population, although they are highly invasive and tumorigenic and the inability of their efficient elimination results in disease relapse and formation of metastases [54]. Fiorillo et al. used flakes of GO to inhibit selectively CSCs proliferation in multiple cell lines including breast, lung, ovarian, prostate, and pancreatic cancers. Two different grades of GO were used, small GO (0,2–2 *μ*m) and big GO (5–20 *μ*m). Both small and big GO flakes inhibited tumor-sphere formation in all independent cancer cells. They did not affect the viability of non-CSCs but selectively targeted cancer stem cells. Analysis of these targeted actions showed that GO inhibited a number of several key signal transduction pathways related to cancer stem cells including antioxidant and interferon responses [55].

## 4. Conclusion

Graphene was first isolated in 2004 and since then its properties have been studied widely [2]. Graphene-based nanomaterials have boosted the development of the interdisciplinary research caused by their unique properties and possible applications in electronics and biotechnology. Single-atom-thick, two-dimensional sheet of sp^2^-hybridized carbon atoms arranged in a regular hexagonal pattern [3, 4] owns extraordinary electrical and thermal properties, mechanical strength, and capability of biofunctionalization [11–13]. Graphene nanoparticles have been used as drug and gene delivery agents in multimodal imaging and could be useful in biomedicine and cancer therapy [20]. Graphene is a nanomaterial whose chemical, physical, or mechanical properties and structure permit the active tissue integration of desirable cell types and tissue components suggesting the potential use in tissue engineering [12, 17, 18]. Besides the research confirming graphene biocompatibility there are reports of dose-dependent graphene toxicity against cultured cells. However, most of these reports concentrate mainly on graphene oxide and reduced graphene oxide (rGO) prepared in solutions [56]. Graphene family nanomaterials include ultrathin graphite, few-layer graphene (FLG), graphene oxide (GO; from monolayer to few layers), reduced graphene oxide (rGO), and graphene nanosheets (GNS) [4]. Among the most frequently used graphene derivatives in the cytotoxicity study are GO, rGO and graphene quantum dots (GQD) with various surface modifications. Mainly studied cancer cells include lung, breast, cervical, liver, and nerve cancer cell lines (Table 1).

Depending on the cell line and type of the nanomaterial, graphene can increase the viability [22, 53] or cause the cell death [33]. In the study of de Marzi et al. GO shows a slight decrease in A549 cells viability while the same concentration and time of exposure result in increased cell viability in CaCo2 colorectal carcinoma cells [22]. Oxidized-graphene nanoribbons (O-GNRs) water-solubilized with the amphiphilic polymer PEG-DSPE (O-GNR-PEG-DSPE) show significantly higher toxic effect on cervical cancer cells (HeLa) than on other cancer or normal tested cells [30]. We can assume that reduced graphene oxide (rGO) is more cytotoxic than graphene oxide (GO) to lung, liver, and breast cancer cells [4, 31, 37]. However, the influence of rGO is similar among U87 nerve cancer cells and MCF-7 breast cancer cell line (IC50 = 85 mg/L and 80 mg/L, resp.) [52]. Graphene surface functionalization with different groups of various biomaterials such as PEG or dextran results in better nanomaterial biocompatibility. Pegylated graphene quantum dots (GQDs-PEG) exhibit very low or no toxicity against lung and cervical cancer cells even at very high concentrations (200 *μ*g/mL) [6, 16]. Pegylated graphene oxide (GO-PEG) [25, 52], dextran covered graphene oxide (GO-DEX) [50] and fluorinated graphene oxide (FGO) [49] are more biocompatible than other graphene derivatives such as highly hydrogenated graphene (HHG) which after 24 h incubation reduce the viability of lung cancer cells (A549) to 26% [24].

Therefore, each graphene derivative may have diverse effect on the same cell type and the same graphene form can cause different reaction depending on the cell origin. Evaluations of the cytotoxicity and biocompatibility are an essential step in developing of any new biomaterial for in vivo biomedical applications. This review reveals that the toxicity of graphene nanomaterials depends not only on the graphene chemical structure, functionalization, size, concentration, and time of exposure but also on various possible mechanisms including interactions with different types of cells or culture medium components. Moreover the diversity of the samples and methods of the production hinder establishing of the biological impact of graphene [21].

One of the proposed mechanisms underlying graphene cytotoxicity involves reactive oxygen species [19, 45] while the others include plasma membrane damage, impairment of mitochondrial activity, DNA damage, and interaction with biomolecules which finally lead to apoptotic and/or necrotic cell death [19, 31, 56]. Toxicity of graphene is desirable when used against cancer cells but not in case of surrounding healthy ones. It would be best to use graphene as a delivery agent for water insoluble drugs, antigens, antibodies, or nucleic acids and unload therapeutic molecules selectively inside the cancer cells to impair their activity [57, 58]. Use of graphene as a drug delivery agent has been recently the subject of numerous scientific researches [7, 12, 17, 23, 25, 27, 29, 38, 39, 41, 58–61]. However, the mechanisms of cellular uptake and modes of action are still under investigation. In vitro studies regarding the influence of GFNs on mammalian cells give only a slight overview on the possible interactions with living organisms. The inconsistency of available data and the lack of sufficient information make it impossible to fully assess the suitability of graphene as a biomaterial. To understand better the impact of graphene further studies should be performed especially in vivo on the mechanisms of cell uptake and signaling combined with the results of long term effects of the materials internalization. More thorough research concerning graphene hemo- and biocompatibility together with the impact on immunological system would be essential to establish safe administration or implantation of GFNs. Yet the most important thing in graphene technology is to establish one universal and recurrent method of production which would allow obtaining graphene with the same properties on large scale and in cost-effective manner. Therefore, detailed studies are required to explain the toxicity pathways of GFNs which would allow not only establishing the effect of graphene on cancer cells but also facilitating their proper use in medicine and cancer therapy.

## Figures and Tables

**Table 1:** Influence of graphene-based nanomaterials on cancer cells.

Cell/tissue	Graphene-based nanomaterials	Dose and time incubation	Percentage of inhibition	Effects	Reference
**MCF-7** breast	**O-GNR-PEG-DSPE** (oxidized-graphene nanoribbons O-GNRs with amphiphilic polymer PEG-DSPE)	10–400 *μ*g/mL 24 h 48 h	24 h: 400 *μ*g/mL = 15% 48 h: 400 *μ*g/mL = 20%	Slight reduction of cell viability	Mullick Chowdhury et al. [30]
	**GO** (1970 nm) **B-rGO** (3833 nm)	0–100 *μ*g/mL 24 h	100 *μ*g/mL = 42% 100 *μ*g/mL = 64%	(i) Reduction of cell viability (ii) ROS generation (iii) Released LDH	Gurunathan et al. [31]
	**GO** (153 nm)	10–80 *μ*g/mL 24 h	80 *μ*g/mL = 13,1%	Reduction of cell viability	Chaudhari et al. [32]

**MDA-MB-231** breast	**GO** (1880 nm) **GE-rGO** (3200 nm)	0–150 *μ*g/mL 24 h 48 h	24 h: 150 *μ*g/mL = 40% 48 h: 150 *μ*g/mL = 50% 24 h: 150 *μ*g/mL = 50% 48 h: 150 *μ*g/mL = 70%	(i) Reduction of cell viability (ii) ROS generation (iii) Released LDH	Gurunathan et al. [5]
	**GO** (156,4 nm)	100–500 *μ*g/mL 48 h	500 *μ*g/mL = 40%	(i) Reduction of cell viability (ii) DNA damage (iii) Interfered with gene expression (iv) Apoptosis	Liu et al. [13]

**SKBR3** breast	**O-GNR-PEG-DSPE** (oxidized-graphene nanoribbons O-GNRs with amphiphilic polymer PEG-DSPE)	10–400 *μ*g/mL 24 h 48 h	24 h: 400 *μ*g/mL = 10% 48 h: 400 *μ*g/mL = 22%	(i) Slight reduction of cell viability (ii) Slight released LDH	Shim et al. [29]

**HeLa** cervix	**GQD – PEG**	0–160 *μ*g/mL 24 h	160 *μ*g/mL = <5%	Slight reduction of cell viability	Chong et al. [16]
	**O-GNR-PEG-DSPE** (oxidized-graphene nanoribbons O-GNRs with amphiphilic polymer PEG-DSPE)	10–400 *μ*g/mL 24 h 48 h	24 h: 400 *μ*g/mL = 60% 48 h: 400 *μ*g/mL = 63%	(i) Reduction of cell viability (ii) Released LDH	Mullick Chowdhury et al. [30]
	**GO** (graphene oxide)	0–80 *μ*g/mL 24 h	80 *μ*g/mL = 50%	(i) Reduction of cell viability (ii) Released LDH (iii) Increased MDA (iv) Decreased SOD (v) ROS generation	Zhang et al. [33]
	**Graphene nanoflakes** 80 nm 30 nm	400 *μ*g/mL 24 h	80 nm: 400 *μ*g/mL = 14% 30 nm: 400 *μ*g/mL = 29%	Reduction of cell viability	Yoon et al. [34]

**A549**	**GQDs** with modified groups NH2, COOH, and CO-N (CH3)2	0–200 *μ*g/mL 24 h	200 *μ*g/mL = <20%	Slight reduction of cell viability	Yuan et al. [6]
	**GO** 1320 nm 130 nm	10; 50; 100 *μ*g/mL 24 h	100 *μ*g/mL = 10%	(i) Slight reduction of cell viability (ii) Genotoxic effect	de Marzi et al. [22]
	**GO** **HHG** (highly hydrogenated graphene)	0–400 *μ*g/mL 24 h	400 *μ*g/mL = 57% 400 *μ*g/mL = 74%	Reduction of cell viability	Chng et al. [24]
	**GO ** s-GO (160 ± 90 nm) m-GO (430 ± 300 nm) l-GO (780 ± 410 nm)	0–200 *μ*g/mL 24 h	s-GO: 200 *μ*g/mL = <33% m-GO, l-GO: 200 *μ*g/mL = <20%	(i) Reduction of cell viability (ii) ROS generation	Chang et al. [35]
	**GO **nanosheets	0–100 *μ*g/mL 24 h	100 *μ*g/mL = 50%	Reduction of cell viability	Hu et al. [36]
	**GO** nanosheets **rGO** nanosheets	20 *μ*g/mL 85 *μ*g/mL 24 h	85 *μ*g/mL = 50% 85 *μ*g/mL = 85%	Reduction of cell viability	Hu et al. [37]

**HepG2** liver	**GO** **rGO**	0-200 *μ*g/mL 24 h	EC20 = 10 *μ*g/mL EC50 = 81 *μ*g/mL EC20 = 8 *μ*g/mL EC50 = 46 *μ*g/mL	(i) Reduction of cell viability (ii) Increased MDA (iii) ROS generation (iv) DNA damage (v) Mitochondrial disorders (vi) Increase in Bax (vii) Decrease in Bcl2	Chatterjee et al. [4]

**U87** nerve	**GPs** (graphene platelets)	0–100 *μ*g/mL 24 h	100 *μ*g/mL = 46%	(i) Reduction of cell viability (ii) Released LDH (iii) Apoptosis	Jaworski et al. [10]
	**GO** 100 nm–10 *μ*m **rGO** 100 nm–1,5 *μ*m	0–100 *μ*g/mL 24 h	100 *μ*g/mL = 28% 100 *μ*g/mL = 64%	(i) Reduction of cell viability (ii) Apoptosis (iii) Reduction of cell proliferation	Jaworski et al. [14]

**U118** nerve	**GPs** (graphene platelets)	0–100 *μ*g/mL 24 h	100 *μ*g/mL = 42%	(i) Reduction of cell viability (ii) Released LDH (iii) Apoptosis	Jaworski et al. [10]
	**GO** 100 nm–10 *μ*m **rGO ** 100 nm–1,5 *μ*m	0–100 *μ*g/mL 24 h	100 *μ*g/mL = 22% 100 *μ*g/mL = 51%	(i) Reduction of cell viability (ii) Apoptosis (iii) Reduction of cell proliferation	Jaworski et al. [14]

**U-138** nerve	**nGr** (nanographene)	50; 100; 250 *μ*g/mL 24 h	250 *μ*g/mL = 35%	Reduction of cell viability	Moore et al. [17]

**RPMI-8226** peripheral blood	**GO**	0–100 *μ*g/mL 24 h	100 *μ*g/mL = 20%	Slight reduction of cell viability	Wu et al. [38]

**PC3** prostate	**CRGO**: chemically reduced graphene oxide	0–180 *μ*g/*μ*L 72 h	160 *μ*g/mL = 60%	Reduction of cell viability	Wang et al. [39]
